# Deprescribing in older adults in a French community: a questionnaire study on patients’ beliefs and attitudes

**DOI:** 10.1186/s12877-024-05165-0

**Published:** 2024-06-27

**Authors:** Thibaut Geremie, Candy Guiguet-Auclair, Marie Laure Laroche, Pierre Mely, Laurent Gerbaud, Marie Blanquet

**Affiliations:** 1 Multi-professional Health Center, Condat, France; 2 Public Health, University Hospital of Clermont-Ferrand, Clermont-Ferrand, France; 3 Clermont Auvergne INP, Clermont Auvergne College, University Hospital of Clermont-Ferrand, CNRS Pascal Institute, Clermont-Ferrand, France; 4 Centre of Pharmacovigilance and Pharmacoepidemiology, Department of Pharmacology- Toxicology and Centre of Pharmacovigilance, University Hospital of Limoges, Limoges, France; 5 UR 24134 (Ageing, Frailty, Prevention, e-Health), Institute Omega Health, University of Limoges, Limoges, France; 6 Surgery of Riom-ès-Montagnes, Riom-ès-Montagnes, France

**Keywords:** Deprescribing, Older adults, Polypharmacy, Primary care

## Abstract

**Background:**

General practitioners (GPs) have a central role to play on reduction of polypharmacy and deprescribing. This study aimed to assess beliefs and attitudes towards deprescribing in patients, aged 65 years or older in primary care, and to identify factors associated with deprescribing and their willingness to stop medication.

**Methods:**

A questionnaire study was performed between 23 May and 29 July 2022 on patients aged 65 years or older attending a GP’s surgery in a French area. We used the French version of the revised Patients’ Attitudes Towards Deprescribing self-report questionnaire (rPATD), which measures four subscales (“Burden”, “Appropriateness”, “Concerns about stopping” and, “Involvement”), patients’ willingness to stop one of their regular medicines, and patients’ satisfaction with their current medicines.

**Results:**

The study enrolled 200 patients. Median age was 76 years old (IQR 71–81), 55% were women, and 42.5% took 5 or more medications per day. Although most patients (92.5%) were satisfied with their current medicines, 35% were reluctant to stop medications they had been taking for a long time, and 89.5% were willing to stop medication if asked to by their GP. Patients aged less than 75 years old reported more concerns about stopping. Women and patients with higher educational attainment showed significantly higher involvement in medication management.

**Conclusions:**

The majority of older adults were willing to stop one or more of their regular medicines if asked to do so by their GP. GPs should address deprescribing into their current practice.

**Supplementary Information:**

The online version contains supplementary material available at 10.1186/s12877-024-05165-0.

## Background

The most approved definition of the deprescribing is that of Reeve et al.: “*Deprescribing is the process of withdrawal of an inappropriate medication, supervised by a health care professional with the goal of managing polypharmacy and improving outcomes”*, where ‘inappropriate medication’ means “*any drug in which the risks outweigh the benefits or […] do not align with goals of care*” [1]. In the French context, a treatment should be necessary and appropriate in order to be relevant [2–4]. Necessary means that the drug should be prescribed according to guidelines, and appropriate means that the drug can be prescribed without harm to the patient. Patients in multimorbid conditions may benefit from a necessary treatment, but may be harmed by the same treatment because it is inappropriate.

Deprescribing is associated with polypharmacy. There is also no single definition of polypharmacy, but the most common in the literature is taking 5 or more medications daily, which is a numerical definition [5, 6]. The pooled prevalence of polypharmacy including all medications, irrespective of the continent, setting and age, was recently reported to be 37% in a meta-analysis and is particularly common in multimorbid older adults [5, 7, 8]. Polypharmacy show increasing trend in developed countries [7–16]. In France, the increasing prevalence of chronic diseases is associated with a higher risk of polypharmacy, with 33–40% of adults aged 75 years old or older taking at least 10 medicines [17, 18]. Polypharmacy has many negative consequences, for which a conceptual classification has been proposed by Wastesson et al. with adverse effects (drug-drug interactions, non-adherence), adverse drug-related events (falls, renal impairment), harm to physical (frailty, sarcopenia) and cognitive function, and hospitalisation and mortality [15, 19–22]. In addition, the pooled prevalence of preventable medication harm was 9% and was higher in older adults (11%), intensive care unit patients (7%) and emergency departments patients (5%) [23]. In adults aged 70 years and older, 10-20% of emergency department admissions are associated with adverse drug reactions [3, 24]. A Cochrane review found that pharmaceutical care can reduce inappropriate prescribing, but there was no convincing evidence of a clinical improvement [25].

General practitioners (GPs) have an essential role to play in preventing polypharmacy and explaining the benefits of withdrawing medicines. However, surveys of GPs’ beliefs in France found that 84% of GPs believed that patients expect to be prescribed a medicine and 62% that patients perceive the discontinuation of a medicine as an abandonment of care [26, 27].

In this context, it is important to explore patients’ beliefs and attitudes towards their medicines, to better understand why polypharmacy remains so prevalent. A previous study was carried out in four French-speaking countries, with a sample of 73 patients in France coming from both community and institutional settings [28]. There is a need to explore the attitudes of French patients towards deprescribing in primary care using a larger sample.

The aim of this study was to analyze beliefs and attitudes towards deprescribing in patients aged 65 years or older in primary care in a French area. The secondary objective was to identify factors associated with attitudes towards deprescribing and willingness to stop medication.

## Methods

### Study design and setting

A questionnaire study was performed in older adults between 23 May and 29 July 2022 in a GP’s surgery in south-central France. The GP had no role in the study design, data collection and analysis. Patients were systematically informed about the study by a research medical student when they came to the surgery for a consultation. They could come for any kind of medical problem. Patients who met the inclusion criteria and who gave their informed consent to participate, completed a self-administered questionnaire after the consultation in a dedicated room next to the waiting room.

### Patients

Patients aged 65 years and older, taking at least one regular medication, living at home, and self-managing their medication (able to prepare and take medication alone, without assistance) were included in the study. Patients were not included if they lived in a nursing home, were unable to self-manage their regular medication, were under legal guardianship, had major cognitive disorders according to the Diagnostic and Statistical Manual of Mental Disorders–Fifth Edition [29], did not speak or read French fluently, or refused to participate.

### Measurements

The revised Australian-validated Patients’ Attitudes Towards Deprescribing questionnaire (rPATD) was used to assess the beliefs and attitudes towards deprescribing in older adults aged 65 years or older [30]. The French version of this self-report questionnaire consists of 20 items grouped into four subscales and two global statements. The fours factors cover “Burden” (perceived burden of medicines, 5 items), “Appropriateness” (beliefs in appropriateness of medications, 5 items), “Concerns about stopping” (5 items), and “Involvement” (in medication management and knowledge of medications, 5 items) [31]. The global statements assess patients’ overall satisfaction about medication (‘Overall, I’m satisfied with my current medicines’) and their willingness to stop medication (‘If my doctor said it was possible, I would be willing to stop one or more of my regular medicines’). Patients responded on a 5-point Likert scale (1 = strongly disagree, 2 = disagree, 3 = unsure, 4 = agree and 5 = strongly agree). Scores for the subscales are obtained by calculating the mean of the individual scores on the items listed in the subscale, with the score for the “Appropriateness” subscale reversed. Higher scores indicate greater perceived burden, belief in appropriateness of current medicines, concerns about stopping a medicine, and involvement in medicine management, willingness to stop medication and satisfaction with current medicines.

Patient age, biological sex, employment status, occupational category before retirement if applicable, level of educational attainment, lifestyle, and number of regular medications were also self-reported. Polypharmacy was defined as taking 5 or more medicines per day.

### Statistical analyses

Categorical variables were expressed as numbers and percentages, and quantitative variables were expressed as median and interquartile range (IQR).

Descriptive analyses were performed to assess sociodemographic and clinical characteristics and the scores on the rPATD subscales.

For the subsequent analyses, all items of the rPATD were dichotomized into those in agreement (agree and strongly agree) and those ambivalent or in disagreement (unsure, disagree and strongly disagree), as done previously [28, 32]. In particular, the global item on willingness to stop medication was converted into a binary variable by combining the response ‘agree’ and ‘strongly agree’ into ‘Willing to stop medication’ and all other responses into ‘Not willing to stop’.

Bivariate analyses were conducted to compare patients who were willing to stop medication versus not willing to stop according to their sociodemographic and clinical characteristics, their scores on rPATD subscales, and their responses to the items of the rPATD subscales (after dichotomization described before). Non-parametric Mann-Whitney tests for quantitative variables and Chi-square or Fisher’s exact tests for categorical variables were used.

To analyze the sociodemographic and clinical factors associated with attitudes and beliefs towards deprescribing, the not normally distributed rPATD subscale scores were dichotomized based on the sample median value, as done previously [28, 32]. They were converted into “low” and “high” score corresponding to a score lower than the median and equal to or higher than the median respectively. Univariate and multivariate logistic regression analyses were performed to investigate which sociodemographic and clinical characteristics (independent variables) affected each separate rPATD subscale score (binary dependent variables). Logistic regressions analyses were performed using a log-binomial distribution, so relative risk (RR) and adjusted relative risk (aRR) were computed with their 95% confidence intervals (95% CI).

Two-sided p-values < 0.05 were considered statistically significant. Statistical analyses were performed using SAS statistics software package v9.4 (SAS Institute Inc., Cary, NC).

### Ethics

The study was approved by the French Committee for the Protection of Individuals southeast 6 (reference 2022/CE37, 31 May 2022) and was conducted in accordance with Declaration of Helsinki principles. All patients gave informed consent to participate.

## Results

### Description of the participant population

During the study period, 202 patients were eligible and all 202 agreed to participate (100%). One participant who did not complete the rPATD and one patient who answered only two items were excluded. Data analyses were therefore conducted on 200 participants. Table 1 reports the socio-demographic and clinical characteristics of the study population. Patients were aged between 65 and 94 years old (58.0% were 75 years and older), and 55% were women. Patients declared taking 1 to 22 medications per day: 42.5% took 5 or more per day, and 11.0% took 10 or more per day.

**Table 1:** Characteristics of the patients and comparison between those who were willing to stop medication and those not willing to stop

	Total	Willing to stop medication ^a^	Not willing to stop medication ^a^	
	*(n = 200)*	*(n = 179, 89.5%)*	*(n = 21, 10.5%)*	*p-value*
**Age** (years old), *median (IQR)*	76 (71–81)	76 (71–82)	77 (72–81)	*0.89*
**Biological sex**, *n (%)*				*0.11*
Women	110 (55.0)	95 (53.1)	15 (71.4)	
Men	90 (45.0)	84 (46.9)	6 (28.6)	
**Occupation before retirement**, *n (%)*				*0.76*
Farmer	43 (21.5)	38 (21.2)	5 (23.8)	
Craftsperson, shopkeeper, business leader	32 (16.0)	29 (16.2)	3 (14.3)	
Managerial role, administrative or professional	48 (24)	41 (22.9)	7 (33.3)	
Employed	40 (20.0)	36 (20.11)	4 (19.0)	
Manual worker / Unemployed	37 (18.5)	35 (19.6)	2 (9.5)	
**Level of educational attainment**, *n (%)*				*0.97*
Low (elementary school)	91 (45.5)	81 (45.3)	10 (47.6)	
Moderate (middle school)	62 (31.0)	56 (31.3)	6 (28.6)	
High (high school and undergraduate)	47 (23.5)	42 (23.5)	5 (23.8)	
**Home-life setting**, *n (%)*				*0.93*
Living alone	65 (32.5)	58 (32.4)	7 (33.3)	
Living with other persons	135 (67.5)	121 (67.6)	14 (66.7)	
**Number of current medications**, *median (IQR)*	5 (3–7)	5 (3–7)	6 (3–8)	*0.65*
**Polypharmacy**, *n (%)*	85 (42.5)	94 (52.5)	13 (61.9)	*0.41*
**rPATD’s subscales scores**^b^, *median (IQR)*				
Burden	2.2 (1.4–2.8)	2.0 (1.4–2.8)	2.2 (1.6–2.8)	*0.37*
Appropriateness	3.8 (3.2–4.4)	3.8 (3.2–4.4)	3.8 (3.2–4.4)	*0.78*
Concerns about stopping	2.2 (1.6–3.0)	2.2 (1.6–2.8)	3.0 (2.6–3.8)	*< 0.001*
Involvement	4.2 (3.8–4.6)	4.2 (3.8–4.6)	4.2 (3.8–4.8)	*0.50*

IQR: interquartile range; Polypharmacy: taking 5 or more medications per day; rPATD: revised patients’ attitudes towards deprescribing

^a^ If patients answered strongly agree or agree to the item “If my doctor said it was possible, I would be willing to stop one or more of my regular medicines”, they were considered to be willing to stop medication. If patients answered unsure, disagree or strongly disagree, they were considered not willing to stop medication

^b^ Possible scores range from 1 to 5. Higher scores indicate greater perceived burden of medication, beliefs in appropriateness, concern about stopping a medicine, and involvement in medication management

### Beliefs and attitudes towards deprescribing

**Fig. 1:**
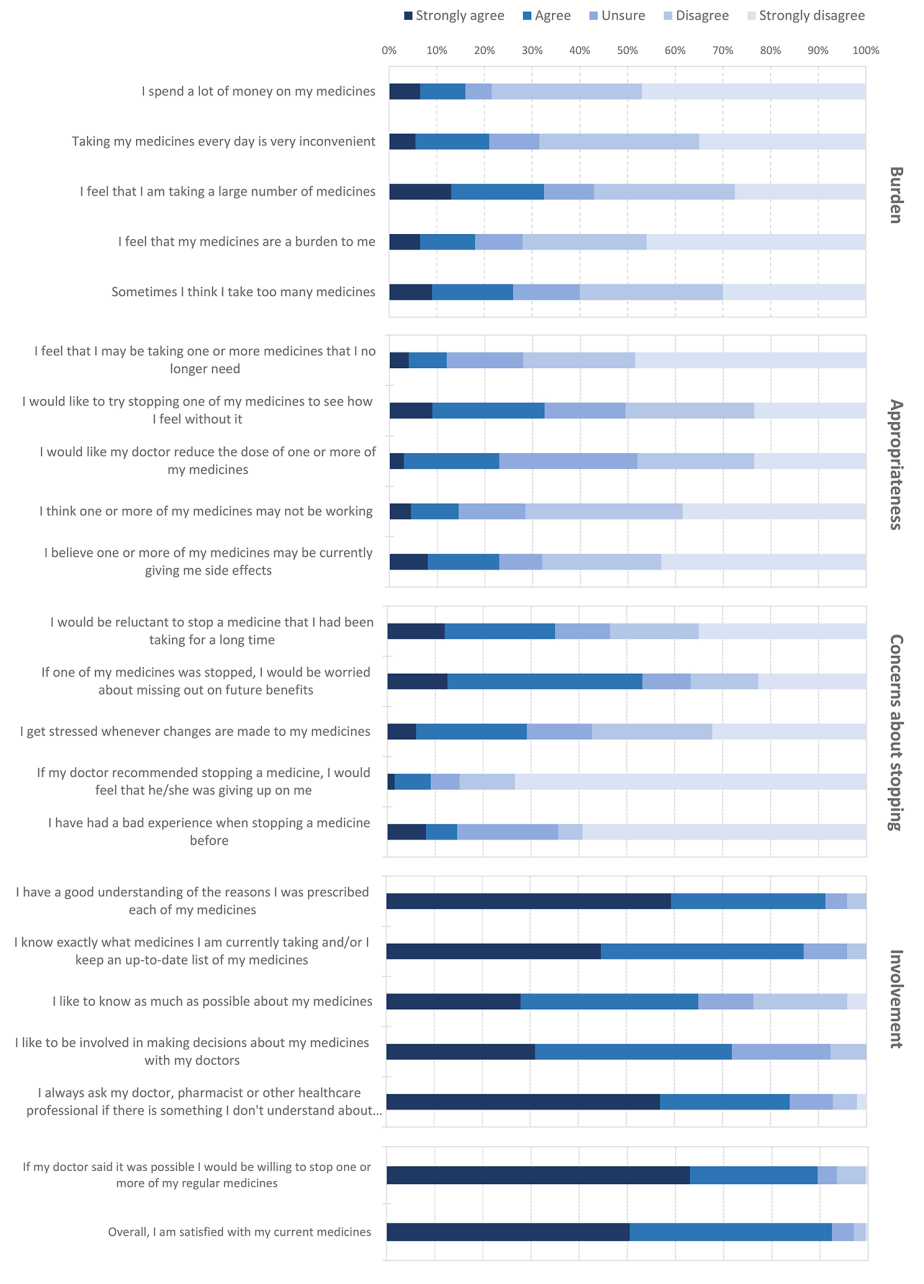
Distribution of the patients’ responses to the rPATD questionnaire

The distribution of the answers to the rPATD items are presented in Fig. 1 and Supplementary Table 1. Patients’ responses to the “Burden” subscale showed that 32.5% agreed that they were taking a large number of medicines (item B3), and 26% thought sometimes that they were taking too many (item B5). The “Concerns about stopping” subscale indicated that 35% reported to be reluctant to stop a medication they had been taking for a long time (item C1), and 53.3% agreed that if one of their medication was stopped, they would be worried about missing out on future benefits (itemC2). Answers to the “Involvement” subscale showed that 91.5% of patients agreed that they had a good understanding of the reasons why they were prescribed each of their medicines (item I1), and 72.0% of patients preferred to be involved in the decision-making process (item I4). The two global statements revealed that 89.5% of patients would be willing to stop one or more medicines if their GP asked them to (item G1), although 92.5% were satisfied with their current medicines (item G2).

The median scores of the rPATD subscales are shown in Table 1. Median scores for “Burden” and “Concerns about stopping” were low (2.2 for each) showing that patients did not feel a high burden and/or high concerns. Median scores for “Appropriateness” and “Involvement” were high (3.8 and 4.2, respectively) showing that patients were convinced that their medication was appropriate and that they understood and managed their medicines well.

### Factors associated with willingness to stop medication

Willingness to stop medication was not associated with any sociodemographic or clinical factors but was significantly associated with the “Concerns about stopping” subscale (*p* < 0.001) (Table 1). Patients who were not willing to stop medication had higher scores, indicating more concerns than those who were willing to stop.

Regarding items of the rPATD, only those on the “Concerns about stopping” subscale, were significantly associated with willingness to stop medication (Supplementary Table 2). Patients who were willing to stop medication were less reluctant to stop a medicine they had been taking for a long time (item C1: 30.7% vs. 71.4%, *p* < 0.001), would be less worried about missing out on future benefits if one their medicine was stopped (item C2: 50.0% vs. 81.0%, *p* = 0.007), would feel less stressed whenever changes are made to their medication (item C3: 27.0% vs. 47.6%, *p* = 0.049), would feel less like the doctor was abandoning them if he/she recommended stopping a medicine (item C4: 7.3% vs. 23.8%, *p* = 0.028), and were less to have had a bad experience when stopping a medicine in the past (item C5: 12.4% vs. 33.3%, *p* = 0.018).

### Factors associated with attitudes towards deprescribing

**Fig. 2:**
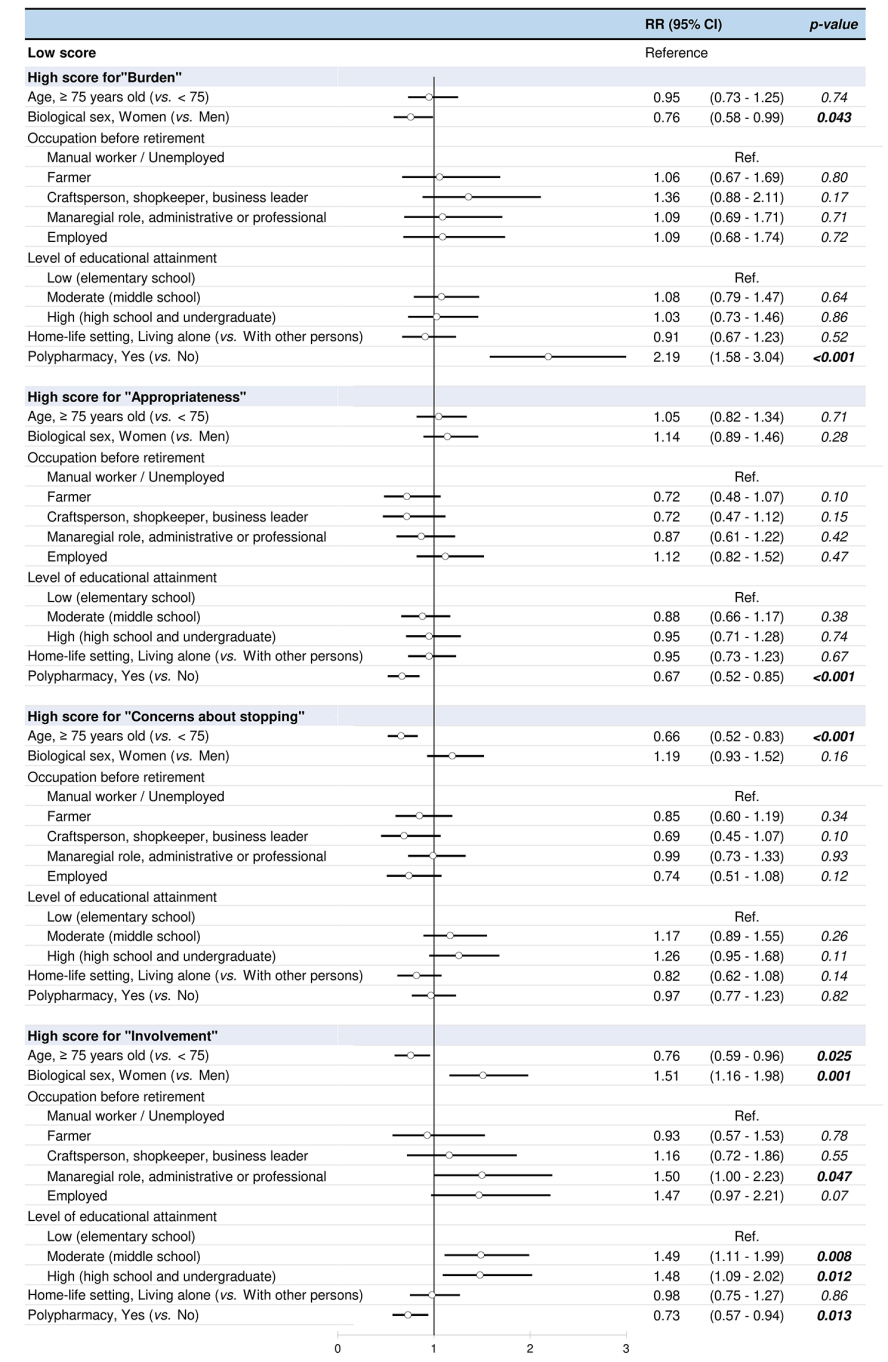
Factors associated with high scores for rPATD subscales in univariate binary logistic regression analysis. High score corresponds to patients with a score for a subscale equal to or higher than the median of the corresponding subscale score. Low score corresponds to patients with a score for a subscale lower than the median of the corresponding subscale score. RR: relative risk; CI: confidence interval; Polypharmacy: taking 5 or more medications per day

We conducted an explorative analysis of potential sociodemographic and clinical factors that could be associated to high score for rPATD subscales. Figure 2 presents the results of the univariate binary logistic regression analysis. This primary analysis showed that men and patients taking 5 or more medications per day were more likely to have high score for the “Burden” subscale. Patients taking 5 or more medications per day were less likely to have high score for the “Appropriateness” subscale. Patients aged 75 years and older were less likely to have high score for the “Concerns about stopping” subscale. Patients aged under 75 years of age, women, patients with a managerial, administrative or professional background, higher level of education and patients taking no more than 5 medications per day were more likely to have high score for the “Involvement” subscale.

In the multivariate analyses, occupation before retirement was not introduced in the model as it was dependent on the level of educational (*p < 0.001*). Multivariate analyses showed that patients taking 5 or more medications per day were at higher risk of having a high score for the “Burden” subscale (greater perceived burden of medication) and a low score for the “Appropriateness” subscale (less beliefs in appropriateness of their medicines). Patients aged 75 years and older were at lower risk of reporting a high score for the “Concerns about stopping” subscales. Women and patients with moderate level of education were at higher risk of reporting high score for the “Involvement” subscale (greater involvement in medication management) Multivariate analyses were displayed in Table 2.

**Table 2:** Multivariate binary logistic regression analysis of high score for rPATD subscales

	aRR (95% CI)	*p*-value
**High score for “Burden”** ^a^		
Age, ≥ 75 years old (vs. < 75)	0.97 (0.77–1.23)	*0.80*
Biological sex, Women (vs. Men)	1.09 (0.85–1.40)	*0.50*
Level of educational attainment		
Low (elementary school)	Ref.	
Moderate (middle school)	1.08 (0.82–1.40)	*0.61*
High (high school and undergraduate)	0.96 (0.70–1.30)	*0.78*
Home-life setting, Living alone (vs. With other persons)	0.84 (0.64–1.10)	*0.21*
Polypharmacy, Yes (vs. No)	**2.20 (1.58–3.08)**	*< 0.001*
**High score for “Appropriateness”** ^a^		
Age, ≥ 75 years old (vs. < 75)	1.04 (0.82–1.33)	*0.75*
Biological sex, Women (vs. Men)	0.93 (0.73–1.18)	*0.55*
Level of educational attainment		
Low (elementary school)	Ref.	
Moderate (middle school)	0.87 (0.66–1.15)	*0.34*
High (high school and undergraduate)	0.96 (0.71–1.28)	*0.76*
Home-life setting, Living alone (vs. With other persons)	0.91 (0.71–1.16)	*0.44*
Polypharmacy, Yes (vs. No)	**0.68 (0.53–0.87)**	*0.002*
**High score for “Concerns about stopping”** ^a^		
Age, ≥ 75 years old (vs. < 75)	**0.69 (0.55–0.87)**	*0.002*
Biological sex, Women (vs. Men)	0.83 (0.66–1.04)	*0.11*
Level of educational attainment		
Low (elementary school)	Ref.	
Moderate (middle school)	1.13 (0.88–1.46)	*0.33*
High (high school and undergraduate)	1.21 (0.92–1.59)	*0.18*
Home-life setting, Living alone (vs. With other persons)	0.86 (0.66–1.12)	*0.26*
Polypharmacy, Yes (vs. No)	1.08 (0.86–1.37)	*0.49*
**High score for “Involvement”** ^a^		
Age, ≥ 75 years old (vs. < 75)	0.84 (0.67–1.07)	*0.16*
Biological sex, Women (vs. Men)	**1.46 (1.11–1.91)**	*0.007*
Level of educational attainment		
Low (elementary school)	Ref.	
Moderate (middle school)	**1.39 (1.04–1.86)**	*0.026*
High (high school and undergraduate)	1.32 (0.97–1.79)	*0.080*
Home-life setting, Living alone (vs. With other persons)	0.96 (0.75–1.23)	*0.77*
Polypharmacy, Yes (vs. No)	0.88 (0.68–1.13)	*0.31*

aRR: adjusted relative risk; CI: confidence interval; Polypharmacy: taking 5 or more medications per day

Bolded values are significant

^a^ High score corresponds to patients with a score for a subscale equal to or higher than the median of the corresponding subscale score. Low score corresponds to patients with a score for a subscale lower than the median of the corresponding subscale score

Higher scores indicate greater perceived burden of medication, beliefs in appropriateness, concern about stopping a medicine, and involvement in medication management

## Discussion

### Main points

A large proportion (89.5%) of our sample were willing to stop one or more of their regular medicines if asked to do so by their GP. Patients who reported a greater willingness to stop medication had fewer concerns about doing so. Conversely, 35% of respondents were reluctant to stop a medicine they had been taking for a long time, and 92.5% were satisfied with their regular medicines. Patients under the age of 75 reported more concerns about stopping a medicine.

### Comparisons with other studies

All previous studies on deprescribing based on the rPATD questionnaire and one based on the beliefs about medicines questionnaire found similar results, with 74.3-97.4% of patients willing to stop one or more of their regular medicines if asked to do so by their GP [28, 32–41]. Two previous meta-analyses- one on 40 trials in 17 countries and one of 29 trials - found that 84% and 87.6% of patients included were willing to stop taking their medicines, respectively [42, 43]. However, there was a difference between low- and middle-income countries and high-income countries: fewer patients in Nepal and Malaysia (< 70%) were willing to stop taking their medicines than in the United States, Australia, and European countries (> 85%) [44]. Previous studies have not clearly identified associated factors such as sociodemographics and number of medications, which means that while polypharmacy warrants regular analysis, it is not sufficient to determine patients’ willingness to stop taking any of their medications [28, 33].The association found here between the “Concern about stopping” subscale and willingness to stop medication has also been shown in two previous studies [28, 32]. The same ambivalence between willingness to stop and reluctance to do so has been found in the literature for people who have been on medication for a long time, making it important to discuss their medication regularly with patients [28, 34, 35, 42].

Several qualitative studies and reviews explored facilitators and barriers to deprescribing in primary care among patients: adults aged 65 years old and older, in adults aged more than 18 years old and frail older adults, carers, healthcare providers and the healthcare system [4, 45–52]. These studies identified that the decision to deprescribe should be: an integral part of the care process, monitored and followed up in coordination with healthcare providers invested in the process, consider the patient as an actor of his/her health through a trusting relationship with healthcare providers. Ambivalence has also been noted between the willingness to stop and the reluctance to stop a medication that they have been taking for a long time and miss the effects of the medication on their health. Studies have shown that healthcare providers have many tools and decisional algorithms at their disposal, but the organisation of care and its fragmentation make it difficult for them to have the time to conduct a comprehensive medical review.

### Strengths and limitations

To our knowledge, this is the first study carried out on deprescribing in primary care in France, where polypharmacy in comorbid older adults is a major issue. This was a monocentric study, so the GP’s behaviour towards medication may be related to his patients ’experience of medication’, which in turns affects the generalizability of the study. The study took place in a practice with patients similar to those seen in other practices in France in terms of multimorbidity but with a larger proportion of older adults (≥ 65 years old). It is the first study in France with a large sample of 200 patients. Nevertheless, the sample size may have been too small to detect some statistically significant differences. Further studies are needed to explore deprescribing in older French patients in a different context (urban area), in all types of patients (needing assistance with medication), and in larger samples.

### Implications for use in clinical practice and research

This study showed that patients are willing to talk to their GPs about deprescribing, which goes against the beliefs of French GPs [26, 27]. Older patients should be targeted first, as they were most at risk of polypharmacy and expressed the least concerns about stopping. Moderate level of education were associated with involvement in medication management, suggesting that further studies should be carried out to analyse the relationship between patients’ health literacy and deprescribing. To our knowledge, none of the qualitative studies identified in the literature were conducted in the French context. It should be relevant to do so in order to identify organisational and health system facilitators and barriers under the prism of the spread of two news developments: advanced practice nurses and territorial health professional communities. Territorial health professional communities have been set up to structure coordination between health professionals on issues relevant to their territory. Polypharmacy should be one of the issues addressed.

## Conclusions

Most patients were willing to stop one or more of their regular medicines if asked to do so by their GP while still being satisfied with their regular medicines. Patients who were less reluctant to stop a medicine they had been taking for a long time were less worried about missing out on future benefits, less stressed about changing their medicines, and less likely to have had a bad experience after stopping a medicine before. Deprescribing appeared to be an area of concern for patients that GPs need to address in their current practice through a consultation dedicated to a comprehensive review of medicines. However, further studies are needed to explore deprescribing in other French contexts with larger samples.

### Electronic supplementary material

Below is the link to the electronic supplementary material.

Supplementary Material 1

## Data Availability

The datasets used and/or analyzed in this study are available from the corresponding author on reasonable request.

## Abbreviations

aRR: Adjusted relative risk
CI: Confidence interval
GP: General practitioner
IQR: Interquartile range
rPATD: Revised Patients’ Attitudes Towards Deprescribing questionnaire
RR: Relative risk
STOPP/START: Screening tool of older people’s prescriptions /screening tool to alert to right treatment
