# Supplementary material for: Deprescribing in older adults in a French community: a questionnaire study on patients’ beliefs and attitudes
Source: BMC Geriatr. 2024 Jun 27;24:562. doi: 10.1186/s12877-024-05165-0 (PMC11212408; doi:10.1186/s12877-024-05165-0)
Supplement: Supplementary file 1 — Supplementary Material 1 [file 12877_2024_5165_MOESM1_ESM.pdf]

Supplementary Table 1: Patients' responses to the rPATD questionnaire

rPATD items		Strongly agree *n (%)*	Agree *n (%)*	Unsure *n (%)*	Disagree *n (%)*	Strongly disagree *n (%)*
**Burden**						
B1	I spend a lot of money on my medicines	13 (6.5)	19 (9.5)	11 (5.5)	63 (31.5)	94 (47.0)
B2	Taking my medicines every day is very inconvenient	11 (5.5)	31 (15.5)	21 (10.5)	67 (33.5)	70 (30.5)
B3	I feel that I am taking a large number of medicines	26 (13.0)	39 (19.5)	21 (10.5)	59 (29.5)	55 (27.5)
B4	I feel that my medicines are a burden to me	13 (6.5)	23 (11.5)	20 (10.0)	52 (26.0)	92 (46.0)
B5	Sometimes I think I take too many medicines	18 (9.0)	34 (17.0)	28 (14.0)	60 (30.0)	60 (30.0)
**Appropriateness**						
A1	I feel that I may be taking one or more medicines that I no longer need	8 (4.0)	16 (8.0)	32 (16.0)	47 (23.5)	97 (48.5)
A2	I would like to try stopping one of my medicines to see how I feel without it	18 (9.0)	47 (23.5)	34 (17.0)	54 (27.0)	47 (23.5)
A3	I would like my doctor reduce the dose of one or more of my medicines	6 (3.0)	40 (20.0)	58 (29.0)	49 (24.5)	47 (23.5)
A4	I think one or more of my medicines may not be working	9 (4.5)	20 (10.0)	28 (14.0)	66 (33.0)	77 (38.5)
A5	I believe one or more of my medicines may be currently giving me side effects	16 (8.0)	30 (15.0)	18 (9.0)	50 (25.0)	86 (43.0)

**Concerns about stopping**						
C1	I would be reluctant to stop a medicine that I had been taking for a long time	24 (12.0)	46 (23.0)	23 (11.5)	37 (18.5)	70 (35.0)
C2	If one of my medicines was stopped, I would be worried about missing out on future benefits	25 (12.6)	81 (40.7)	20 (10.1)	28 (14.1)	45 (22.6)
C3	I get stressed whenever changes are made to my medicines	12 (6.0)	46 (23.1)	27 (13.6)	50 (25.1)	64 (32.2)
C4	If my doctor recommended stopping a medicine, I would feel that he/she was giving up on me	3 (1.5)	15 (7.5)	12 (6.0)	23 (11.6)	146 (73.4)
C5	I have had a bad experience when stopping a medicine before	16 (8.0)	13 (6.5)	42 (21.1)	10 (5.0)	118 (59.3)
**Involvement**						
I1	I have a good understanding of the reasons I was prescribed each of my medicines	118 (59.3)	64 (32.2)	9 (4.5)	8 (4.0)	0
I2	I know exactly what medicines I am currently taking and/or I keep an up-to-date list of my medicines	89 (44.7)	84 (42.2)	18 (9.1)	8 (4.0)	0
I3	I like to know as much as possible about my medicines	56 (28.0)	74 (37.0)	23 (11.5)	39 (19.5)	8 (4.0)
I4	I like to be involved in making decisions about my medicines with my doctors	62 (31.0)	82 (41.0)	41 (20.5)	15 (7.5)	0

I5	I always ask my doctor, pharmacist or other healthcare professional if there is something I don't understand about my medicines	114 (57.0)	54 (27.0)	18 (9.0)	10 (5.0)	4 (2.0)
**General statements**						
G1	If my doctor said it was possible I would be willing to stop one or more of my regular medicines	126 (63.0)	53 (26.5)	8 (4.0)	12 (6.0)	1 (0.5)
G2	Overall, I am satisfied with my current medicines	101 (50.5)	84 (42.0)	9 (4.5)	5 (2.5)	1 (0.5)

rPATD: revised patients' attitudes towards deprescribing.

Supplementary Table 2: Comparison of the rPATD items between those who were willing to stop medication and those not willing to stop

rPATD items [b]			**Willing to stop medication** [a] *(n=179, 89.5%)*	**Not willing to stop medication** [a] *(n=21, 10.5%)*	*p-value*
**Burden**					
B1	I spend a lot of money on my medicines	Agree	28 (15.6)	4 (19.0)	*0.75*
		Disagree	151 (84.4)	17 (81.0)	
B2	Taking my medicines every day is very inconvenient	Agree	37 (20.7)	5 (23.8)	*0.78*
		Disagree	142 (79.3)	16 (76.2)	
B3	I feel that I am taking a large number of medicines	Agree	57 (31.8)	8 (38.1)	*0.56*
		Disagree	122 (68.2)	13 (61.9)	
B4	I feel that my medicines are a burden to me	Agree	34 (19.0)	2 (9.5)	*0.38*
		Disagree	145 (81.0)	19 (90.5)	
B5	Sometimes I think I take too many medicines	Agree	46 (25.7)	6 (28.6)	*0.78*
		Disagree	133 (74.3)	15 (71.4)	
**Appropriateness**					
A1	I feel that I may be taking one or more medicines that I no longer need	Agree	23 (12.8)	1 (4.8)	*0.48*
		Disagree	156 (87.2)	20 (95.2)	
A2	I would like to try stopping one of my medicines to see how I feel without it	Agree	59 (33.0)	6 (28.6)	*0.68*
		Disagree	120 (67.0)	15 (71.4)	

A3	I would like my doctor reduce the dose of one or more of my medicines	Agree	43 (24.0)	3 (14.3)	*0.42*
		Disagree	136 (76.0)	18 (85.7)	
A4	I think one or more of my medicines may not be working	Agree	24 (13.4)	5 (23.8)	*0.20*
		Disagree	155 (86.6)	16 (76.2)	
A5	I believe one or more of my medicines may be currently giving me side effects	Agree	44 (24.6)	2 (9.5)	*0.17*
		Disagree	135 (75.4)	19 (90.5)	
**Concerns about stopping**					
C1	I would be reluctant to stop a medicine that I had been taking for a long time	Agree	55 (30.7)	15 (71.4)	*<0.001*
		Disagree	124 (69.3)	6 (28.6)	
C2	If one of my medicines was stopped, I would be worried about missing out on future benefits	Agree	89 (50.0)	17 (81.0)	*0.007*
		Disagree	89 (50.0)	4 (19.0)	
C3	I get stressed whenever changes are made to my medicines	Agree	48 (27.0)	10 (47.6)	*0.049*
		Disagree	130 (73.0)	11 (52.4)	
C4	If my doctor recommended stopping a medicine, I would feel that he/she was giving up on me	Agree	13 (7.3)	5 (23.8)	*0.028*
		Disagree	165 (92.7)	16 (76.2)	
C5	I have had a bad experience when stopping a medicine before	Agree	22 (12.4)	7 (33.3)	*0.018*
		Disagree	156 (87.6)	14 (66.7)	
**Involvement**					
I1	I have a good understanding of the reasons I was prescribed each of my medicines	Agree	161 (90.4)	21 (100)	*0.23*
		Disagree	17 (9.6)	0	
I2		Agree	153 (86.0)	20 (95.2)	*0.32*

	I know exactly what medicines I am currently taking and/or I keep an up-to-date list of my medicines	Disagree	25 (14.0)	1 (4.8)	
I3	I like to know as much as possible about my medicines	Agree	115 (64.2)	15 (71.4)	*0.51*
		Disagree	64 (35.8)	6 (28.6)	
I4	I like to be involved in making decisions about my medicines with my doctors	Agree	128 (71.5)	16 (76.2)	*0.65*
		Disagree	51 (28.5)	5 (23.8)	
I5	I always ask my doctor, pharmacist or other healthcare professional if there is something I don't understand about my medicines	Agree	152 (84.9)	16 (76.2)	*0.34*
		Disagree	27 (15.1)	5 (23.8)	

rPATD: revised patients' attitudes towards deprescribing.

[a] If patients answered strongly agree or agree to the item “If my doctor said it was possible, I would be willing to stop one or more of my regular medicines”, they were considered to be willing to stop medication. If patients answered unsure, disagree or strongly disagree, they were considered not willing to stop medication.

[b] All items of the rPATD were dichotomized into “agree” (agree and strongly agree) and “disagree” (unsure, disagree and strongly disagree).
